# Anaesthesia Management of Laparoscopic Subtotal Gastrectomy in a Patient With Atrial Myxoma: A Case Report

**DOI:** 10.7759/cureus.70144

**Published:** 2024-09-25

**Authors:** Lee Shiuan Tay, Chi Ho Chan, Xuanxuan Chen

**Affiliations:** 1 Department of Anaesthesiology, Sengkang General Hospital, Singapore, SGP

**Keywords:** atrial myxoma, gastrectomy, general anaesthesia, intraoperative complications, laparoscopy

## Abstract

Atrial myxoma is a rare cardiac tumour that is associated with serious complications such as sudden cardiac death and stroke and warrants early surgical resection. We report a case of a 73-year-old male with an incidental diagnosis of left atrial myxoma undergoing general anaesthesia for laparoscopic subtotal gastrectomy, D2 lymphadenectomy, and Roux-en-Y gastroduodenectomy for a newly diagnosed pyloric tumour. Careful anaesthetic considerations and management need to be taken when undergoing non-cardiac surgery to mitigate the peri-operative complications of the left atrial myxoma. Collaborative management under a multidisciplinary team of anaesthetists, surgeons, cardiologists, and cardiothoracic surgeons is essential.

## Introduction

Atrial myxoma is a type of rare primary cardiac tumour with an incidence ranging from 0.0017% to 0.02% and is surgically excised as soon as possible once diagnosed in view of potential complications including sudden cardiac death [1,2]. In the current literature, patients with atrial myxoma who underwent non-cardiac surgery are limited to case reports as most patients would have had early surgical resection of the atrial myxoma.

In this report, we present the case of a patient with left atrial myxoma who underwent non-cardiac surgery under general anaesthesia (GA) for laparoscopic subtotal gastrectomy, D2 lymphadenectomy, and Roux-en-Y gastroduodenectomy in view of a pyloric tumour with gastric outlet obstruction.

This case report was prepared according to the CARE guidelines for case reports [3]. This article was presented as a poster at the KoreAnesthesia 2023 conference, November 9-11, 2023.

## Case presentation

A 73-year-old Chinese male with a newly diagnosed pyloric tumour was electively scheduled for laparoscopic subtotal gastrectomy, D2 lymphadenectomy, and Roux-en-Y gastroduodenectomy under GA. A cleaner by occupation, he weighed 62 kg and had a height of 1.58 m. He was an ex-smoker with a 30-pack-year smoking history and his medical history included hyperlipidaemia, gout, and paroxysmal atrial fibrillation for which he was not on anticoagulation therapy. He had a previous uneventful incision and drainage of left knee abscess under GA. He presented with a two-month history of intermittent abdominal pain, abdominal distension with bloating, and nausea. An abdominal-computed tomography (CT) of the abdomen and pelvis was performed and revealed the presence of a pyloric tumour, explaining his symptoms which were consistent with gastric outlet obstruction. A naso-jejunal tube was inserted while awaiting surgical excision of the tumour.

**Figure 1:**
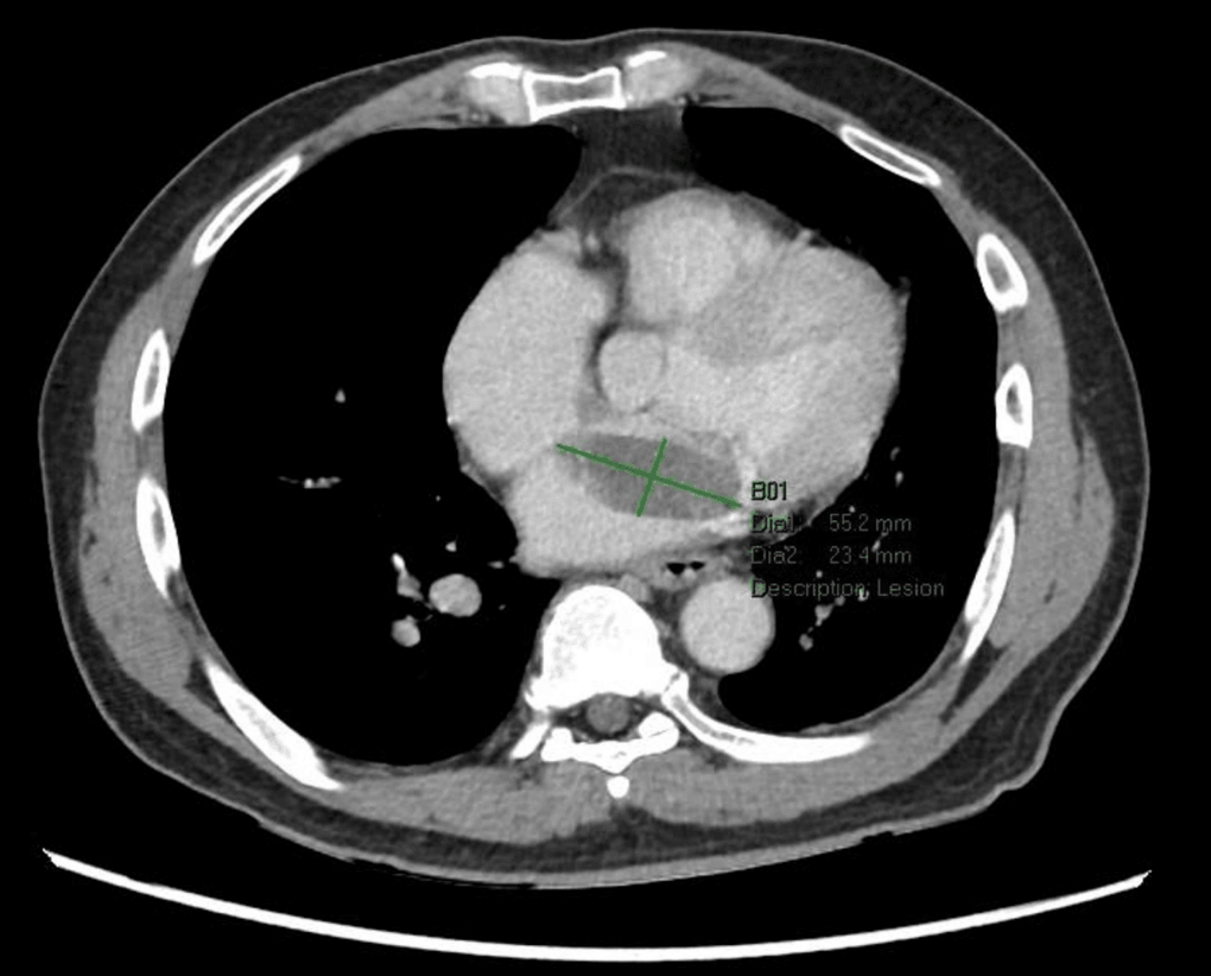
Transverse CT image of the lower thorax showing an incidental finding of a large filling defect in the left atrium with its two-dimensional measurement indicated in green.

**Figure 2:**
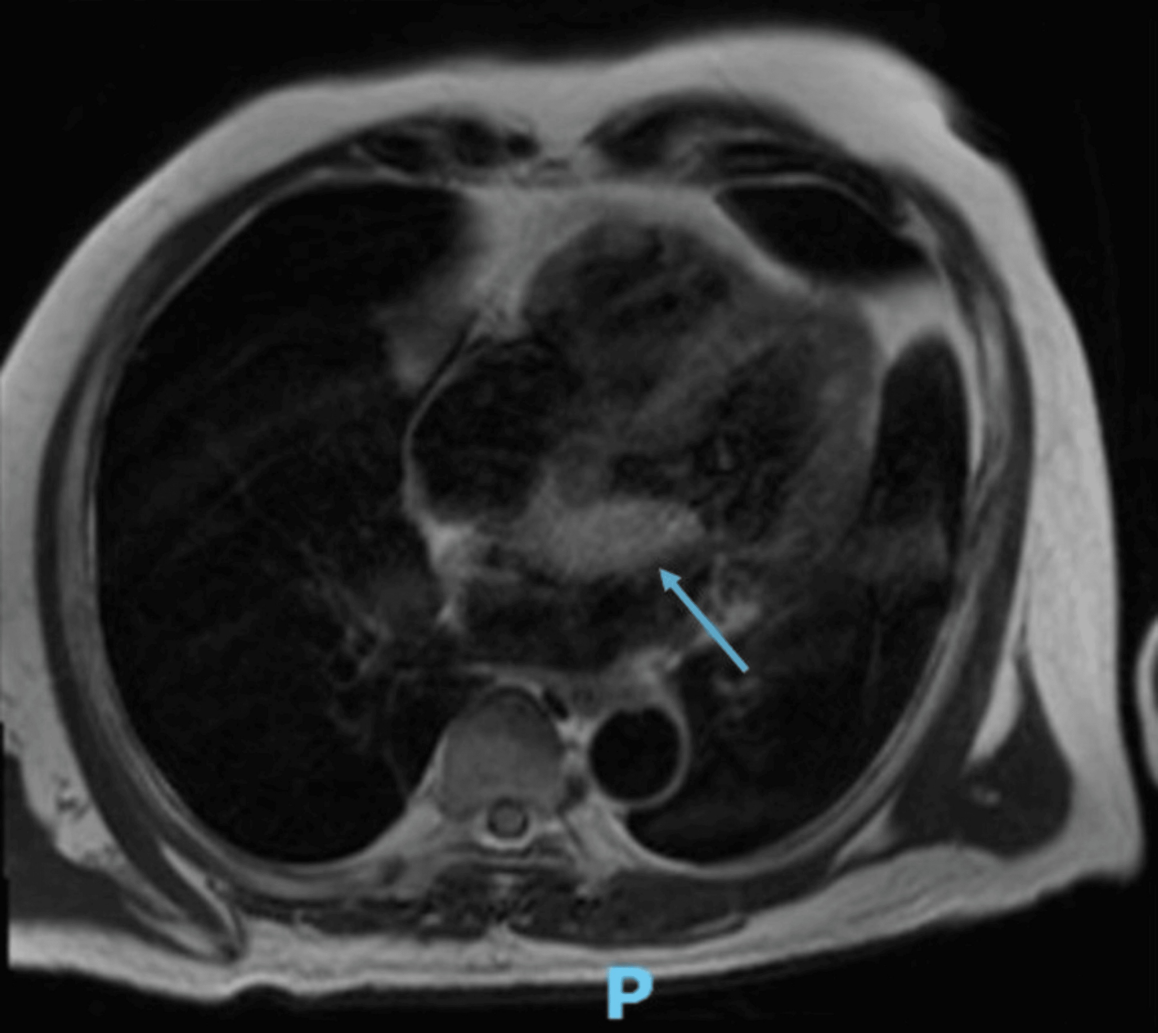
Cardiac MRI image in T2 sequence. The blue arrow indicates the location of a narrow-based left atrial mass measuring 4.3 x 1.9 cm arising from the interatrial septum.

This case was complicated by an incidental finding of a left atrial myxoma, where a large filling defect in the left atrium was captured during abdominal CT imaging (Figure 1). A transthoracic echocardiogram (TTE) was performed to further evaluate this filling defect, revealing a hyperechoic spherical structure attached to interatrial septum, measuring 44 mm x 17 mm, abutting the anterior mitral leaflet in diastole (Video 1). This was suggestive of an atrial myxoma. TTE also reported a left ventricular ejection fraction of 62% with no regional wall motion abnormalities noted. A cardiac magnetic resonance imaging (MRI) was performed after consultation with a cardiologist, confirming the diagnosis of an atrial myxoma (Figure 2).

**Video 1:** Transthoracic echocardiogram four-chamber view showing a hyperechoic spherical structure attached to interatrial septum and abutting the anterior mitral leaflet in diastole.

A multi-disciplinary discussion from general surgery, cardiology, cardiothoracic surgery, and anaesthesiology was conducted to discuss whether excision of atrial myxoma needs to precede laparoscopic subtotal gastrectomy. The concerns about proceeding with laparoscopic subtotal gastrectomy before atrial myxoma excision include an elevated risk of major adverse cardiac events and an elevated risk of stroke from myxoma embolisation. However, proceeding with atrial myxoma excision first may result in a delay of tumour resection and potential metastatic spread of the tumour. On preoperative assessment, the patient was haemodynamically stable with good cardiac function. There was an absence of audible cardiac murmurs, and the electrocardiogram findings were unremarkable. Physical examination was unremarkable. As the patient was asymptomatic from the atrial myxoma, the decision was made to delay the resection of the atrial myxoma and proceed with the subtotal gastrectomy.

On the day of surgery, he was transferred to the operating room for the planned surgery. To ensure adequate cardiac preload, 500 ml of Hartmann’s solution was administered prior to induction of anaesthesia. Standard monitoring used included non-invasive blood pressure, electrocardiogram, and pulse oximetry. In addition, a left radial arterial line was placed for close monitoring of blood pressure, and a right internal jugular vein central venous catheter for monitoring of central venous pressure (CVP) and administration of inotropes and vasopressors. SEDLine® Brain Monitoring (Masimo Corporation, Irvine, California, United States) was used to monitor and titrate the depth of anaesthesia. O3® Regional Oximetry (Masimo Corporation) was placed over both hemispheres to monitor brain perfusion and for early detection of stroke. Defibrillator pads were also placed prior to induction with a resuscitation trolley on standby in the operating room.

The patient was pre-oxygenated with 6 L of 100% oxygen prior to induction. A rapid sequence induction was performed using propofol 80 mg, fentanyl 100 mcg, and suxamethonium 100 mg. A size 8 Portex® Profile Soft Seal® Cuff (Portex Inc., San Francisco, California, United States) endotracheal tube was inserted using a size 3 McGrath^TM^ MAC video laryngoscope (Medtronic plc, Minneapolis, Minnesota, United States) with first-pass success. Anaesthesia was maintained with sevoflurane, remifentanil, and atracurium throughout the operation. In terms of ventilation, our patient was on pressure-controlled volume-guaranteed mode throughout the surgery to lower the required ventilatory pressure. A total of 3000 ml of Hartmann's solution was administered during the operation and blood loss was 200 ml. Peri-operatively, his blood pressure was maintained within 20% of his preoperative mean arterial pressure using a total of 1.2 mg of phenylephrine and 30mg of ephedrine. Morphine was administered towards the end of the operation for postoperative analgesia. Cerebral regional oximetry values ranged from 60 to 74 during the entire surgery with no significant change from pre-induction values. The effect of neuromuscular blockade was reversed with glycopyrrolate 0.4 mg and neostigmine 2.5 mg and the patient was extubated uneventfully at the end of surgery. The duration of surgery was five hours and 50 minutes.

The patient remained stable in the post-anaesthetic care unit and was subsequently discharged to a high dependency unit for closer monitoring in the immediate postoperative period. He was prescribed intravenous paracetamol and a patient-controlled intravenous morphine pump for postoperative pain control. His recovery was complicated by pneumonia triggering episodes of atrial fibrillation with rapid ventricular rate, in which cardiology was consulted and treated with intravenous digoxin and oral bisoprolol. He also experienced delayed gastric emptying attributed to a kink in the Roux limb of the gastrojejunostomy, which was managed with dilation via oesophagogastroduodenoscopy. He was discharged in stable condition three weeks after the surgery and planned for outpatient coronary evaluation prior to further management of the left atrial myxoma. An oncology review was also scheduled to consider adjuvant chemotherapy for the histologically confirmed gastric adenocarcinoma.

## Discussion

In patients presenting with multiple concurrent urgent surgical pathologies at the same time, choosing which surgery to prioritise can be a challenging decision. Complications of untreated atrial myxoma include embolic complications and sudden death due to mitral valve obstruction or coronary valve obstruction, with a reported mortality of up to 8% while waiting for surgical intervention [1,2,4]. However, delaying resection of the gastric tumour may also worsen gastric obstruction symptoms and risk the progression of malignancy. Multi-disciplinary discussion with the relevant subspecialties will be essential to understand the risks and benefits of each option to make the most appropriate decision for our patient. In our case, given that our patient had asymptomatic atrial myxoma with gastric outlet obstruction from a pyloric tumour, the decision from the multi-disciplinary discussion was for the subtotal gastrectomy to precede the atrial myxoma surgery.

Left atrial myxoma is a benign cardiac tumour that may present with symptoms of mitral valve obstruction, peripheral embolism (stroke or embolism to peripheries), or systemic symptoms such as fever or loss of weight [2,4]. Cardiac myxoma can be asymptomatic in 20% of patients and presents as an incidental finding, but those with mitral valve obstruction may present with shortness of breath, weakness, pulmonary oedema, syncope, or sudden death [4,5]. It is imperative to perform a thorough pre-operative assessment and cardiac investigations in the presence of a cardiac tumour. Echocardiography can provide information about the size and location of the tumour but may not confidently differentiate between a cardiac myxoma and a cardiac thrombus [6]. Cardiac MRI allows accurate diagnosis and measurement and provides information about the functional impact of the masses to guide further management of such lesions [6]. Urgent surgery for atrial myxoma may be warranted in the event of acute symptomatic presentation such as heart failure with pulmonary oedema, signs of myxoma embolism, or based on echocardiogram findings of tumour prolapsing into the mitral orifice or a large tumour with an elevated risk of embolism to relieve symptoms and prevent further complications of atrial myxoma [7]. However, in stable and asymptomatic patients, non-urgent cardiac surgery can allow for a more comprehensive pre-operative assessment and optimisation. This may include treating pulmonary oedema with supplemental oxygen and non-invasive ventilation, optimisation of baseline heart rate with beta-blockers, and optimisation of intravascular fluid status.

Anaesthetic considerations for patients with left atrial myxomas undergoing GA are similar to those for patients with mitral stenosis, mimicking a fixed cardiac output physiology [8]. Furthermore, low atrial volume may induce saddling of the myxoma onto the mitral valve, worsening the mitral outflow obstruction [9]. As such, proper optimisation of fluid status prior to surgery is important, especially in the setting of an atrial myxoma. The optimal haemodynamic goals would include (i) maintaining adequate preload to maintain atrial volume, (ii) optimising filling of the left ventricle by maintaining sinus rhythm to preserve the atrial kick and avoiding tachycardia to allow for a longer diastolic time, (iii) maintaining right ventricular contractility while minimising pulmonary vascular resistance to optimise right-to-left cardiac blood flow, and (iv) maintaining systemic afterload to ensure adequate prefusion pressure to the organs despite reduced cardiac output [10,11]. The placement of an arterial catheter to allow close monitoring of haemodynamic stability and a central venous catheter to allow monitoring of CVP to guide intraoperative fluid management may be useful. In our case, a transoesophageal echocardiogram was not utilised but could be useful for intraoperative monitoring of atrial myxoma position to detect potential embolism of myxoma and guide fluid management.

Laparoscopic intra-abdominal surgery requires the generation of pneumoperitoneum with carbon dioxide for surgical visualisation leading to a rise in intra-abdominal pressure. This can compress the inferior vena cava resulting in a reduction in cardiac preload. Additionally, cephalad displacement of the diaphragm may cause an increase in intra-thoracic pressure, reducing the cardiac chamber wall compliance. Laparoscopic gastrectomy surgery often entails steep reverse Trendelenburg positioning which results in a reduction in venous return. In patients with mitral stenosis-like physiology, these physiological changes may be unfavourable. However, laparoscopic surgery reduces postoperative operative site pain and discomfort as compared to open surgery, which reduces the incidence of significant tachycardia and may be favourable for patients with atrial myxoma. Furthermore, laparoscopic surgery results in shorter recovery time, and lower incidence of wound infection as compared to open surgery [12]. The choice between laparoscopic and open surgery may be inconclusive in this situation and should be evaluated on a case-by-case basis. If open surgery were to be the surgical approach of choice, epidural analgesia may be warranted during the perioperative period for perioperative pain management and avoidance of tachycardia.

Among existing literature, several case reports detail patients with atrial myxoma who underwent surgery under anaesthesia. We did not identify any reports of patients with atrial myxoma undergoing laparoscopic general surgery. Reshma et al. highlighted a patient with left atrial myxoma who underwent right-hand wound debridement under nerve block to avoid the complications of GA and to maintain stable haemodynamic status [13]. Ture et al. discussed a case of a patient with left atrial myxoma who underwent GA for repair of epigastric hernia with administration of titrated doses of anaesthetics and an epidural block to minimise haemodynamic fluctuations [8]. Arumugam et al. discussed a case of a 25-year-old woman at 29 weeks gestation with a large left atrial myxoma undergoing GA for an early elective lower segment caesarean section to avoid the sudden decrease in afterload after subarachnoid block that may precipitate intracardiac obstruction by the myxoma [14]. The patient subsequently underwent an excision of the atrial myxoma the next day under GA and cardiopulmonary bypass. Singh et al. presented a case report of a patient with left atrial myxoma who underwent radical mastectomy for a left breast tumour under GA with the goals of ensuring blood pressure and heart rate were close to resting state to ensure adequate atrial volume [9]. In these case reports, the outcomes were largely uneventful.

## Conclusions

Effective management of non-cardiac surgery in the presence of an atrial myxoma requires a collaborative approach involving a multidisciplinary team of anaesthetists, surgeons, cardiologists, and cardiothoracic surgeons. This is crucial for assessing the risks and benefits of the available options to decide on the most appropriate management option. It is essential to engage the patient and their family in these discussions to ensure a collaborative, informed decision.
